# Methyl 2,5-dichloro­benzoate

**DOI:** 10.1107/S1600536808029541

**Published:** 2008-09-20

**Authors:** Tariq Mahmood Babar, Ghulam Qadeer, Nasim Hasan Rama, Ales Ruzicka, Zdenka Padelkova

**Affiliations:** a Department of Chemistry, Quaid-i-Azam Univeristy, Islamabad 45320, Pakistan; b Department of General and Inorganic Chemistry, Faculty of Chemical Technology, University of Pardubice, Nam. Cs. Legii’ 565, 53210 Pardubice, Czech Republic

## Abstract

In the mol­ecule of the title compound, C_8_H_6_Cl_2_O_2_, the benzene ring is oriented with respect to the planar ester group at a dihedral angle of 39.22 (3)°.

## Related literature

For general background, see: Zheng *et al.* (2003 ▶); Al-Talib *et al.* (1990 ▶); Yousif *et al.* (1986 ▶); Ahmad *et al.* (2001 ▶); Al-Soud *et al.* (2004 ▶); El-Emam *et al.* (2004 ▶); Weinstock *et al.* (1991 ▶). For a description of the Cambridge Structural Database, see: Allen (2002 ▶); and of *MOGUL, see: *Bruno *et al.* (2004 ▶). 
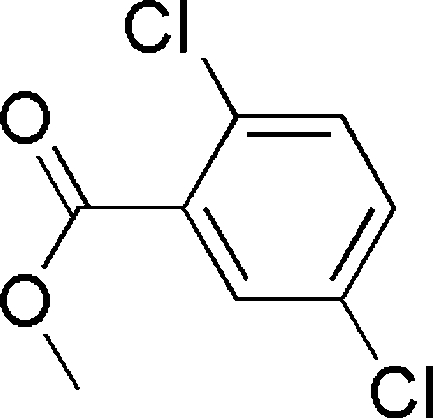


## Experimental

### 

#### Crystal data

C_8_H_6_Cl_2_O_2_
*M* *_r_* = 205.03
Triclinic, 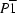

*a* = 3.8452 (3) Å
*b* = 7.0158 (4) Å
*c* = 15.8510 (10) Å
α = 77.189 (6)°
β = 89.130 (7)°
γ = 83.741 (5)°
*V* = 414.46 (5) Å^3^
*Z* = 2
Mo *K*α radiation
μ = 0.73 mm^−1^
*T* = 150 (1) K
0.68 × 0.11 × 0.06 mm

#### Data collection

Bruker–Nonius Kappa CCD area-detector diffractometer
Absorption correction: Gaussian (Coppens, 1970 ▶) *T*_min_ = 0.864, *T*_max_ = 0.971
5966 measured reflections
1840 independent reflections
1455 reflections with *I* > 2σ(*I*)
*R*_int_ = 0.110

#### Refinement

*R*[*F* ^2^ > 2σ(*F* ^2^)] = 0.053
*wR*(*F* ^2^) = 0.146
*S* = 1.09
1840 reflections
109 parameters
H-atom parameters constrained
Δρ_max_ = 0.44 e Å^−3^
Δρ_min_ = −0.57 e Å^−3^

### 

Data collection: *COLLECT* (Hooft, 1998 ▶)and *DENZO* (Otwin­owski & Minor, 1997 ▶); cell refinement: *DIRAX/LSQ* (Duisenberg, 1992) ▶); data reduction: *EvalCCD* (Duisenberg, 1992) ▶); program(s) used to solve structure: *SIR92* (Altomare *et al.*, 1994 ▶); program(s) used to refine structure: *SHELXL97* (Sheldrick, 2008 ▶); molecular graphics: *PLATON* (Spek, 2003 ▶); software used to prepare material for publication: *SHELXL97*.

## Supplementary Material

Crystal structure: contains datablocks I. DOI: 10.1107/S1600536808029541/hk2533sup1.cif

Structure factors: contains datablocks I. DOI: 10.1107/S1600536808029541/hk2533Isup2.hkl

Additional supplementary materials:  crystallographic information; 3D view; checkCIF report

## Figures and Tables

**Fig. 1.**
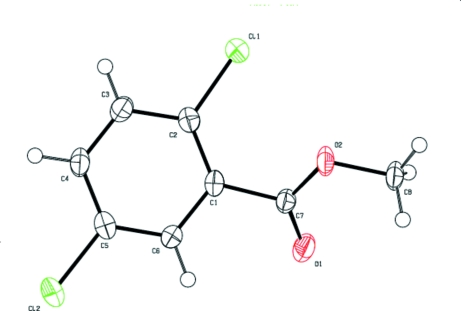
The molecular structure of the title molecule, with the atom-numbering scheme. Displacement ellipsoids are drawn at the 50% probability level.

**Fig. 2.**
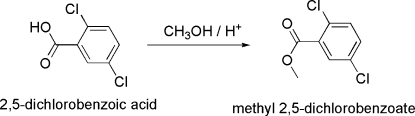
Reaction scheme.

## supplementary crystallographic information

### Comment

The title compound is a lachrymator and a drug intermediate. Methyl 2,5-di- chlorobenzoate is widely employed in synthetic organic chemistry for example, 2,5-dichlorobenzohydrazide, 2,5-disubstituted-1,3,4-oxadiazoles (Zheng *et al.*,  2003; Al-Talib *et al.*, 1990) and 5-substituted-2-mercapto-1,3,4-oxadiazoles (Yousif *et al.*,  1986; Ahmad *et al.*,  2001; Al-Soud *et al.*, 2004; El-Emam *et al.*, 2004). In addition, methyl 4-(bromomethyl)benzoate has been used in the synthesis of 1-(carboxybenzyl)imidazole-5-acrylic acids, which are potent and selective angiotensin II receptor antagonists (Weinstock *et al.*, 1991).In view of the versatility of these compounds, we have synthesized the title compound, and report herein its crystal structure.

In the molecule of the title compound, (Fig. 1), the bond lengths and angles are generally within normal ranges (Cambridge Structural Database, Version 5.28, November 2006; Mogul Version 1.1; Allen, 2002, Bruno *et al.*,  2004). The benzene ring (C1-C6) is oriented with respect to the planar ester group (O1/O2/C1/C7/C8) at a dihedral angle of 39.22 (3)°.

### Experimental

For the preparation of the title compound, the mixture of 2,5-dichlorobenzoic acid (2.05 g, 10 mmol) and absolute methanol (50 ml) in the presence of a few drops of suphuric acid was refluxed for 5 h. The excess of solvent was removed by distillation. The solid residue for filltered off, washed with water and recystallized from ethanol (30%) to give the title compound (yied; 88%, m.p. 319-321 K). Suitable single crystals of the title compound were obtained by slow evaporation of an ethanol solution at room temperature.

### Refinement

H atoms were positioned geometrically, with C-H = 0.93 and 0.96 Å for aromatic and methyl H, respectively, and constrained to ride on their parent atoms with U_iso_(H) = 1.2U_eq_(C).

### Crystal data

**Table:**

C_8_H_6_Cl_2_O_2_	*Z* = 2
*M**_r_* = 205.03	*F*(000) = 208
Triclinic, *P*1	*D*_x_ = 1.643 Mg m^−^^3^
Hall symbol: -P 1	Melting point: 319(2) K
*a* = 3.8452 (3) Å	Mo *K*α radiation, λ = 0.71073 Å
*b* = 7.0158 (4) Å	Cell parameters from 6024 reflections
*c* = 15.851 (1) Å	θ = 1–27.5°
α = 77.189 (6)°	µ = 0.73 mm^−^^1^
β = 89.130 (7)°	*T* = 150 K
γ = 83.741 (5)°	Needle, colorless
*V* = 414.46 (5) Å^3^	0.68 × 0.11 × 0.06 mm

### Data collection

**Table:**

Bruker–Nonius KappaCCD area-detector diffractometer	1840 independent reflections
Radiation source: fine-focus sealed tube	1455 reflections with *I* > 2σ(*I*)
graphite	*R*_int_ = 0.110
Detector resolution: 9.091 pixels mm^-1^	θ_max_ = 27.5°, θ_min_ = 2.6°
φ and ω scans	*h* = −4→4
Absorption correction: gaussian (Coppens, 1970)	*k* = −9→8
*T*_min_ = 0.864, *T*_max_ = 0.971	*l* = −20→20
5966 measured reflections	

### Refinement

**Table:**

Refinement on *F*^2^	Primary atom site location: structure-invariant direct methods
Least-squares matrix: full	Secondary atom site location: difference Fourier map
*R*[*F*^2^ > 2σ(*F*^2^)] = 0.053	Hydrogen site location: inferred from neighbouring sites
*wR*(*F*^2^) = 0.146	H-atom parameters constrained
*S* = 1.10	*w* = 1/[σ^2^(*F*_o_^2^) + (0.0527*P*)^2^ + 0.5264*P*] where *P* = (*F*_o_^2^ + 2*F*_c_^2^)/3
1840 reflections	(Δ/σ)_max_ < 0.001
109 parameters	Δρ_max_ = 0.44 e Å^−^^3^
0 restraints	Δρ_min_ = −0.57 e Å^−^^3^

### Special details

**Table:**

Geometry. All e.s.d.'s (except the e.s.d. in the dihedral angle between two l.s. planes) are estimated using the full covariance matrix. The cell e.s.d.'s are taken into account individually in the estimation of e.s.d.'s in distances, angles and torsion angles; correlations between e.s.d.'s in cell parameters are only used when they are defined by crystal symmetry. An approximate (isotropic) treatment of cell e.s.d.'s is used for estimating e.s.d.'s involving l.s. planes.
Refinement. Refinement of *F*^2^ against ALL reflections. The weighted *R*-factor *wR* and goodness of fit *S* are based on *F*^2^, conventional *R*-factors *R* are based on *F*, with *F* set to zero for negative *F*^2^. The threshold expression of *F*^2^ > σ(*F*^2^) is used only for calculating *R*-factors(gt) *etc*. and is not relevant to the choice of reflections for refinement. *R*-factors based on *F*^2^ are statistically about twice as large as those based on *F*, and *R*- factors based on ALL data will be even larger.

### Fractional atomic coordinates and isotropic or equivalent isotropic displacement parameters (Å^2^)

**Table:**

	*x*	*y*	*z*	*U*_iso_*/*U*_eq_	
Cl1	0.6965 (2)	0.52269 (12)	0.70713 (5)	0.0309 (2)	
Cl2	1.3585 (2)	−0.28182 (13)	0.92310 (5)	0.0361 (3)	
O1	0.6439 (7)	−0.0150 (4)	0.62680 (14)	0.0347 (6)	
O2	0.8542 (6)	0.2744 (4)	0.57949 (13)	0.0288 (5)	
C1	0.9066 (8)	0.1286 (5)	0.72871 (18)	0.0220 (6)	
C2	0.8841 (8)	0.2952 (5)	0.76262 (19)	0.0235 (6)	
C3	1.0100 (9)	0.2857 (5)	0.8458 (2)	0.0278 (7)	
H3	0.9954	0.3983	0.8680	0.033*	
C4	1.1574 (9)	0.1080 (5)	0.89485 (19)	0.0286 (7)	
H4	1.2435	0.1006	0.9501	0.034*	
C5	1.1755 (8)	−0.0580 (5)	0.8613 (2)	0.0257 (7)	
C6	1.0489 (8)	−0.0510 (5)	0.77933 (19)	0.0245 (6)	
H6	1.0581	−0.1648	0.7581	0.029*	
C7	0.7825 (8)	0.1206 (5)	0.64006 (18)	0.0229 (6)	
C8	0.7421 (9)	0.2749 (5)	0.49289 (19)	0.0294 (7)	
H8A	0.8509	0.1593	0.4761	0.035*	
H8B	0.8089	0.3898	0.4536	0.035*	
H8C	0.4924	0.2757	0.4914	0.035*	

### Atomic displacement parameters (Å^2^)

**Table:**

	*U*^11^	*U*^22^	*U*^33^	*U*^12^	*U*^13^	*U*^23^
Cl1	0.0411 (5)	0.0249 (4)	0.0250 (4)	0.0029 (3)	−0.0044 (3)	−0.0047 (3)
Cl2	0.0429 (5)	0.0328 (5)	0.0276 (4)	0.0027 (4)	−0.0115 (3)	0.0014 (3)
O1	0.0496 (16)	0.0344 (14)	0.0231 (11)	−0.0124 (11)	−0.0066 (10)	−0.0089 (10)
O2	0.0368 (13)	0.0354 (13)	0.0137 (10)	−0.0082 (10)	−0.0056 (8)	−0.0017 (9)
C1	0.0214 (15)	0.0287 (16)	0.0156 (13)	−0.0036 (12)	−0.0005 (10)	−0.0039 (11)
C2	0.0233 (15)	0.0256 (16)	0.0205 (14)	−0.0043 (12)	−0.0010 (11)	−0.0021 (12)
C3	0.0331 (18)	0.0295 (18)	0.0230 (15)	−0.0050 (13)	−0.0007 (12)	−0.0096 (13)
C4	0.0336 (18)	0.0348 (18)	0.0172 (14)	−0.0055 (14)	−0.0045 (12)	−0.0042 (12)
C5	0.0233 (16)	0.0303 (17)	0.0206 (14)	−0.0028 (12)	−0.0014 (11)	0.0003 (12)
C6	0.0302 (17)	0.0236 (16)	0.0205 (14)	−0.0008 (12)	−0.0026 (11)	−0.0073 (12)
C7	0.0257 (15)	0.0265 (16)	0.0166 (13)	0.0004 (12)	−0.0013 (11)	−0.0062 (11)
C8	0.0367 (19)	0.0368 (19)	0.0141 (13)	0.0003 (14)	−0.0047 (12)	−0.0061 (12)

### Geometric parameters (Å, °)

**Table:**

Cl1—C2	1.728 (3)	C3—H3	0.9300
Cl2—C5	1.737 (3)	C4—C3	1.382 (5)
O1—C7	1.199 (4)	C4—C5	1.378 (5)
O2—C7	1.326 (4)	C4—H4	0.9300
O2—C8	1.444 (3)	C5—C6	1.384 (4)
C1—C2	1.385 (5)	C6—H6	0.9301
C1—C6	1.395 (4)	C8—H8A	0.9600
C1—C7	1.505 (4)	C8—H8B	0.9600
C2—C3	1.396 (4)	C8—H8C	0.9600
			
C7—O2—C8	115.5 (3)	C4—C5—Cl2	119.7 (2)
C2—C1—C6	119.3 (3)	C6—C5—Cl2	118.9 (3)
C2—C1—C7	125.6 (3)	C5—C6—C1	119.3 (3)
C6—C1—C7	115.1 (3)	C5—C6—H6	120.4
C1—C2—Cl1	123.1 (2)	C1—C6—H6	120.2
C1—C2—C3	120.7 (3)	O1—C7—O2	124.7 (3)
C3—C2—Cl1	116.3 (3)	O1—C7—C1	122.4 (3)
C4—C3—C2	119.7 (3)	O2—C7—C1	112.9 (3)
C4—C3—H3	120.1	O2—C8—H8A	109.4
C2—C3—H3	120.3	O2—C8—H8B	109.5
C5—C4—C3	119.5 (3)	H8A—C8—H8B	109.5
C5—C4—H4	120.3	O2—C8—H8C	109.5
C3—C4—H4	120.2	H8A—C8—H8C	109.5
C4—C5—C6	121.4 (3)	H8B—C8—H8C	109.5
